# Angioleiomyoma of the Volar Aspect of the Right Small Finger: A Case Report

**DOI:** 10.7759/cureus.89165

**Published:** 2025-07-31

**Authors:** Sahil Dave, Suhirad Khokhar, Waqas Mahmud, Mohamed Alshal, John Diks

**Affiliations:** 1 Pathology, Marshall University Joan C. Edwards School of Medicine, Huntington, USA; 2 Orthopedics, Marshall University Joan C. Edwards School of Medicine, Huntington, USA

**Keywords:** angioleiomyoma of the finger, painful nodule, soft tissue tumor, subcutaneous mass, surgical excision

## Abstract

A 53-year-old right-hand-dominant man presented with a recurrent, painful mass localized to the volar surface of the right small finger, leading to discomfort and impaired function. Radiographic imaging was unremarkable for osseous involvement. Surgical excision followed by histopathological and immunohistochemical (IHC) analysis established the diagnosis of angioleiomyoma, marked by desmin-positive smooth muscle fibers and CD34-positive vasculature. Given its infrequency in the hand and overlap with more common benign lesions, angioleiomyoma remains diagnostically elusive. This report emphasizes the pivotal role of tissue-based diagnostics and supports excision as a definitive treatment modality.

## Introduction

Angioleiomyoma is a benign neoplasm composed of well-differentiated smooth muscle cells originating from the tunica media of vascular walls [1]. Although these tumors commonly affect the lower extremities, particularly in middle-aged women, their presence in the hand is uncommon, comprising less than 1% of all soft tissue tumors in the upper extremity [2,3]. Proposed etiologies include hormonal influences, localized trauma, or vascular dysfunction, though these remain speculative [4]. 

Patients typically present with a well-circumscribed, slowly enlarging nodule, often associated with tenderness due to nerve involvement or vascular spasm [5,6]. In atypical locations such as the hand, the tumor may clinically resemble more prevalent entities such as ganglion cysts, lipomas, or soft tissue sarcomas, contributing to frequent misdiagnoses [3,7]. 

While imaging modalities such as ultrasound and MRI can assist in lesion characterization, findings are frequently nonspecific [5,8]. Thus, histologic and immunohistochemical (IHC) examinations remain essential for accurate diagnosis. Positive staining for smooth muscle markers including desmin and smooth muscle actin (SMA), in conjunction with vascular endothelial markers such as CD34, supports the diagnosis. Lack of reactivity to neural and histiocytic markers (e.g., S100, SOX10, CD68) helps distinguish angioleiomyomas from other spindle cell neoplasms [6,9]. 

This case highlights a diagnostically challenging presentation of angioleiomyoma in an unusual anatomic location and underscores the importance of histopathologic confirmation. 

## Case presentation

A 53-year-old right-hand-dominant man with a medical history of treated Hodgkin’s lymphoma, attention-deficit/hyperactivity disorder (ADHD), and radiation-induced heart disease presented for evaluation of a painful, intermittently enlarging mass on the volar aspect of his right fifth digit for several months. The lesion fluctuated in size and was aggravated by manual activities. He noted a temporary reduction after self-manipulation but with consistent recurrence. No accident or trauma was associated with the onset of his symptoms. 

**Figure 1:**
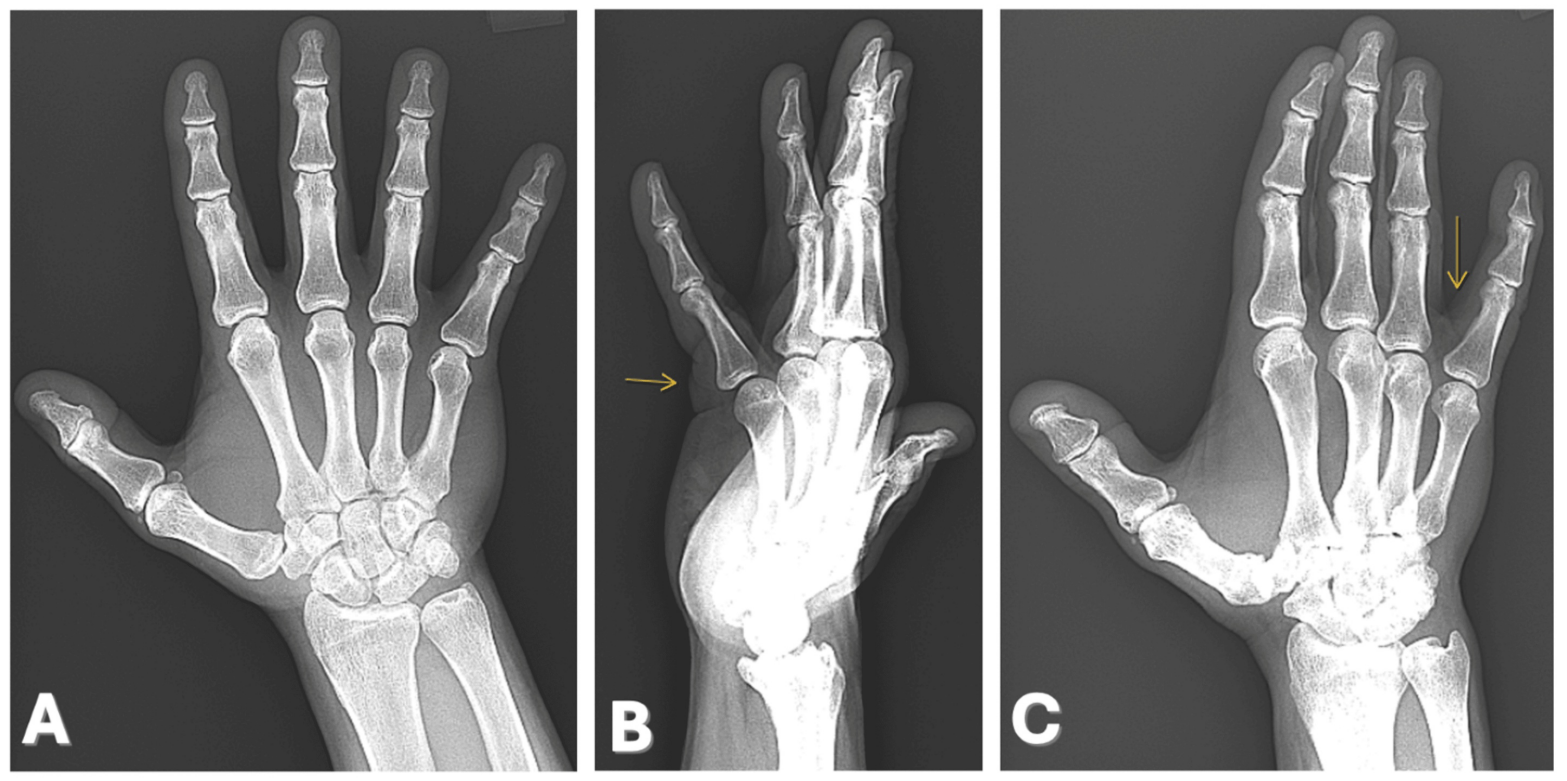
X-rays of the right hand showing no significant osseous abnormalities or erosions involving the phalanges or metacarpal of the small finger. (A) Anterior-posterior, (B) lateral, and (C) oblique views. Arrows: location of the lesion.

Examination revealed a firm, mobile, tender subcutaneous nodule without overlying skin changes. Radiographs of the right hand demonstrated no bony abnormalities or erosive features (Figure 1). 

Surgical excision was performed under local anesthesia. The lesion was located over the flexor tendon sheath at the proximal phalanx (P1) in zone 2 of the small finger. The cyst was separated from the surrounding neurovascular bundle. Grossly, the excised specimen measured 1.0 × 0.6 × 0.4 cm and appeared as a well-circumscribed, tan-brown fibrotic nodule with a homogeneously firm cut surface. 

**Figure 2:**
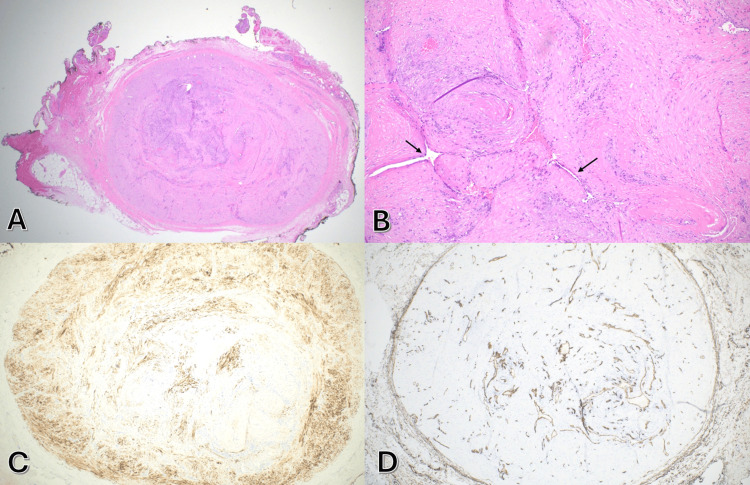
(A) Hematoxylin and eosin (H&E) stain (x20) demonstrating a well-demarcated, encapsulated nodule of bland spindle cells in a whorled arrangement. (B) H&E (x100) revealing bundles of smooth muscle surrounding vascular lumens (arrows) without cytologic atypia or mitotic activity. (C) Desmin immunohistochemical (IHC) stain (x40) demonstrating diffuse, robust cytoplasmic positivity in the smooth muscle. (D) CD34 IHC (x40) showing endothelial staining in numerous vascular channels.

Microscopically, the lesion was composed of uniform spindle cells concentrically arranged around numerous small blood vessels, consistent with a vascular smooth muscle neoplasm. At a higher magnification, a whorled pattern of smooth muscle bundles surrounding vascular spaces was observed (Figures 2A, 2B). 

IHC analysis showed strong cytoplasmic desmin positivity in the spindle cells, and CD34 highlighted the endothelial lining of intralesional vessels (Figures 2C, 2D). Markers for neural and histiocytic origin including S100, SOX10, and CD68 were negative, confirming the diagnosis of angioleiomyoma and ruling out peripheral nerve sheath tumors and fibrohistiocytic lesions. 

Postoperatively, the patient experienced complete symptomatic relief and regained normal digit function. No recurrence was observed at clinical follow-up. 

## Discussion

Due to their rarity in the hand and nonspecific clinical presentation, angioleiomyomas are commonly mistaken for more prevalent benign lesions such as ganglion cysts or giant cell tumors of the tendon sheath. Patients commonly report long-standing swelling with a gradual increase in size. It usually presents as a small, firm, mobile subcutaneous nodule with well-defined margins. Pain is a frequent symptom and may be spontaneous or triggered by pressure or cold exposure [2-4,10]. Cases involving the digital or ulnar arteries highlight that angioleiomyoma may arise directly from vascular walls, leading to pulsatility or tenderness along arterial paths [4,11]. This diagnostic ambiguity often delays definitive treatment and underscores the importance of tissue analysis. 

Histologically, angioleiomyomas consist of interlacing bundles of smooth muscle surrounding vascular lumens and are classified into solid, venous, or cavernous subtypes [1,9]. The solid subtype, often associated with pain, is most frequently encountered in clinical practice. 

While imaging may suggest a vascular-rich soft tissue lesion, only histopathology in conjunction with immunohistochemistry can establish a definitive diagnosis. Immunoprofiling is critical in differentiating angioleiomyoma from leiomyosarcoma, schwannoma, or other spindle cell neoplasms [6,9]. In this case, the combination of desmin and CD34 positivity with the absence of S100 and SOX10 expression was diagnostic. 

Complete surgical excision remains the treatment of choice. The prognosis is excellent, with recurrence rates below 5% following complete resection and no known malignant transformation [2,4,7]. Our patient's postoperative course mirrored those outcomes, with full functional recovery and no lesion recurrence. 

## Conclusions

This case illustrates a diagnostically challenging presentation of angioleiomyoma in the hand, an uncommon location for this tumor type. The lesion’s clinical mimicry of more common entities underscores the importance of maintaining a broad differential diagnosis for digital masses. Histopathological examination with IHC correlation remains indispensable for accurate diagnosis. Complete surgical excision offers a curative outcome with low recurrence risk. 
